# Progressive damage along the optic nerve following induction of crush injury or rodent anterior ischemic optic neuropathy in transgenic mice

**Published:** 2008-11-30

**Authors:** O. Dratviman-Storobinsky, M. Hasanreisoglu, D. Offen, Y. Barhum, D. Weinberger, N. Goldenberg-Cohen

**Affiliations:** 1 Department of Ophthalmology, Rabin Medical Center, Petah Tiqwa, Israel; 2 The Krieger Eye Research Laboratory, Felsenstein Medical Research Center, Tel Aviv University, Petah Tiqwa, Israel; 3 Neuroscience Laboratory, Felsenstein Medical Research Center, Tel Aviv University, Petah Tiqwa, Israel; 4 The Mina and Everard Goodman Faculty of Life Sciences, Bar Ilan University, Ramat Gan, Israel; 5 Sackler School of Medicine, Tel Aviv University, Tel Aviv, Israel; 6 Department of Ophthalmology, Pediatric Ophthalmology Unit, Schneider Children's Medical Center of Israel, Petah Tiqwa, Israel

## Abstract

**Purpose:**

To characterize the histological changes that occur in response to induction of ischemic or mechanical optic nerve damage in transgenic mice.

**Methods:**

Either optic nerve crush injury or rodent anterior ischemic optic neuropathy (rAION) were induced in the right eye of mice transgenic for the *Thy1* gene promoter expressing cyan fluorescent protein (CFP; n=40) and mice transgenic for the cyclic nucleotide phosphodiesterase (CNPase) gene promoter expressing green fluorescent protein (GFP; n=40). The left eye served as a control. The mice were euthanized at different times after injury. Eyes were enucleated, and the brain together with the optic nerves was completely dissected. Cryopreserved sections of both optic nerves were analyzed by fluorescence microscopy. In addition, flat-mounted retinas from the Thy1-CFP mice were analyzed for retinal ganglion cell (RGC) loss.

**Results:**

Axonal loss was detected in the right eye of the Thy1-CFP mice, and demyelination was detected in the CNPase-GFP mice. Both processes occurred simultaneously in the two models of injury. The damage proceeded retrogradely and, in the crush-injury group, crossed the chiasm within 4 days. At 21 days after injury, RGC loss measured 70% in the crush-injury group and 25% in the rAION group.

**Conclusions:**

Axonal injury and demyelination along the optic nerves occur simultaneously in transgenic mice exposed to ischemic or crush injury. The degree of RGC loss reflects the severity of the injury. Loss of oligodendrocytes and myelin apparently leads to axonal loss. Transgenic mice offer a promising model for exploring the damage caused by optic nerve injury. Use of fluorescence labeling makes it possible to better understand the underlying pathophysiology, which can help researchers formulate neuroprotective agents.

## Introduction

Optic nerve injury causes severe axonal damage leading to apoptosis of the retinal ganglion cells (RGCs) and consequent loss of vision [1-3]. The mechanisms of axonal damage have been examined in different animal models: optic nerve transection and crush [4-6]; acute retinal ischemia and reperfusion [7-9]; chronic elevation of intraocular pressure (IOP) [10-12]; and rose-bengal-photoactivation-induced anterior ischemic optic neuropathy (AION) [12,13].

Studies in mice transgenic for the *c-fos* gene have reported that the induction of AION in rodent led to overall axonal loss, with variations in the severity of RGC loss in the retina from region to region [14]. Progressive oligodendrocyte stress and demyelination were detected along the injured optic nerve [13]. These findings suggested that in optic nerve injury, RGC loss and axonal involvement occur simultaneously with oligodendrocyte stress, which proceeds rapidly through the chiasm [13]. Oligodendrocytes are known to be essential for neuronal cell body and axonal survival as well as for myelin assembly [15]. Given that a single oligodendrocyte myelinates many axons [15,16], under ischemic conditions demyelination may lead to additional loss of function also in axons in the proximity of the injury and to additional RGC death. A blockage in axonal transportation as well may further increase the damage and RGC death. At the same time, the RGC loss itself can cause axonal degeneration with associated oligodendrocyte dysfunction and consequent demyelination. It is not yet clear whether it is the loss of the oligodendrocytes that causes the axonal breakdown or if the axonal loss leads to oligodendrocyte death, or both. These axonal and myelin changes have not been examined specifically in labeled transgenic mice at different intervals after injury.

Thy1, a surface glycoprotein, is uniquely expressed by ganglion cells in the retina [17] and has been found to serve as a useful marker of RGC loss and axonal damage. Studies of optic nerve injury have shown a decrease in expression of the *Thy1* gene or a depletion of the Thy1 protein following optic nerve damage [18], indicating early stress to the RGCs [18-20]. However, these studies did not elaborate on the association of axonal loss with demyelination.

The 2',3′-cyclic nucleotide 3′-phosphodiesterase enzyme (CNPase) is involved in myelin synthesis and is present in high levels in brain and peripheral nerves [21]. It is found almost exclusively in the myelin-producing oligodendrocyte cells in the central nervous system. Therefore, CNPase can be used as a marker for oligodendrocyte integrity [22]. Reduced CNPase levels have been reported in various neurologic and demyelinating diseases [23,24]. The aim of the present study was to further characterize the pathophysiologic processes underlying the axonal and myelin optic nerve changes in response to injury of differing severity in transgenic Thy1 and CNPase mice.

## Methods

### Animals

All protocols were conducted in accordance with the ARVO Statement for the Use of Animals in Ophthalmic and Vision Research and were approved and monitored by the Animal Care Committee of Rabin Medical Center, Petah Tiqwa, Israel. The animals were housed under a 14 h:10 h light-dark cycle with standard chow and water ad libitum.

All mice included in the study (n=100; 80 transgenic, 20 nontransgenic for apoptosis assay) were at least 2 months old and weighed more than 25 g. We induced crush injury in 40 mice and rodent anterior ischemic optic neuropathy (rAION) in 40 mice. All mice were analyzed accordingly. Findings were evaluated in transgenic Thy1 mice labeled for cyan fluorescent protein (CFP; Thy1-CFP transgenic mice were kindly provided by Dr. Steven Bernstein, University of Maryland, Baltimore, MD) and transgenic CNPase mice, labeled for green fluorescent protein (GFP; CNPase-GFP transgenic mice were obtained from Dr. Vittorio Gallo, Center for Neuroscience Research, Children's Research Institute, Washington, D.C.). Transgenes were verified by polymerase chain reaction using gene-specific primers and genomic DNA.

### Induction of crush injury

Using 80 mg/kg ketamine and 4 mg/kg xylazine, we anesthetized 20 Thy1-CFP and 20 CNPase-GFP transgenic mice. We then crushed the right optic nerve in each mouse by applying forceps at 2.5–3.0 mm posterior to the globe for 7 s; this procedure was performed 3 times. The left eyes were not treated and served as controls. Subsets of mice in each group were euthanized by carbon dioxide (CO2) asphyxiation at 1, 3, 4, 7, 14, and 21 days after injury. The eyes were enucleated and the brain together with the optic nerves was completely dissected. Cryopreserved sections of brain, chiasm, and both optic nerves were analyzed by fluorescence microscopy. In addition, flat-mounted retinas from the Thy1-CFP mice were analyzed for RGC loss.

### Induction of rAION

rAION was induced as previously described [13]. In brief, 20 mice from each group were anesthetized with 80 mg/kg ketamine and 4 mg/kg xylazine, and the right pupils were dilated with eyedrops of 0.25% phenylephrine hydrochloride and 0.5% tropicamide (Mydramid). A custom-designed, plastic fundus, corneal contact lens was used for direct visualization of the retina and optic nerve head. After intravenous administration of 0.05 ml of 2.5 mM rose bengal in phosphate-buffered saline (PBS X1, Biological Industries, Ltd., Kibbutz Beit Haemek, Israel), the right optic nerve head was illuminated with argon green laser (532 nm, 200 µm spot size, 50 mW power) for 0.1 s. The left eyes did not undergo laser activation and served as controls. Subsets of animals were euthanized at 1, 3, 4, 7, 14, and 21 days after rAION induction. The eyes were enucleated, and the brain together with the optic nerves was completely dissected. Cryopreserved sections of both optic nerves were analyzed by fluorescence microscopy. In addition, flat-mounted retinas of the Thy1-CFP mice were analyzed for RGC loss.

### Apoptosis assay

Longitudinal cross-sections of the cryopreserved eyes and optic nerves were cut 6 µm thick for in situ TdT-mediated dUTP nick end labeling assay (TUNEL; Roche, Mannheim, Germany). Staining was performed with the fluorescein-tagged apoptosis detection system. Hoechst stain was used to identify nuclear changes. Results were analyzed with a fluorescence microscope (Fluoview X; Olympus, Tokyo, Japan) at 580 nm wavelength. The mean number of TUNEL-positive cells per slide was determined in consecutive sections; special attention was addressed to the specific retinal layer. Findings were compared between the study eyes and control eyes at the different time points and between the models of optic nerve injury.

### Cell counting

The number of RGCs in the Thy1-CFP mice was quantified by ImageJ, a public domain Java image processing program (developed at the National Institutes of Health, Washington, D.C.). To verify the findings, we stained retinal flat mounts with Hoechst nuclear dye to examine nuclei in the RGC layer. Under fluorescence microscopy, the RGC number was assessed by averaging the RGC counts from at least six fields that were equidistant from the optic nerve.

We also used ImageJ to determine the number of oligodendrocyte cells in the optic nerve of the CNPase-GFP mice. Three different sites, proximal to distal (from the globe to the chiasm), were analyzed. In the axons, we were unable to quantify the labeled RGC loss by direct calculation, so we estimated the loss relative to the control nerve using the transgenic fluorescence labeling of the axons.

The total loss of labeled axons in the Thy1-CFP mice was analyzed according to the final RGC loss measured in the retinas. The findings were compared between injured and control eyes at various time points after the induction of injury.

## Results

### Crush model

#### Thy1-CFP transgenic mice

**Figure 1:**
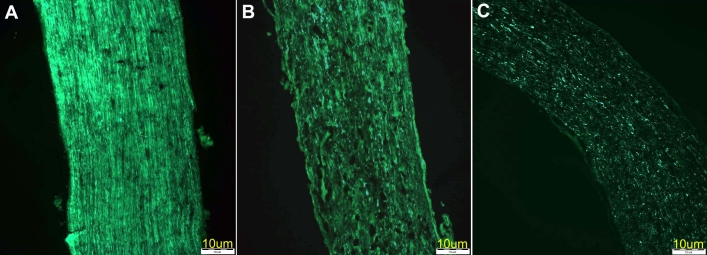
Axonal loss in optic nerves of Thy1-CFP mice at various intervals after crush injury. **A:** Control optic nerve; no damage. Note the absence of damage to the optic nerve. **B:** Four days after crush injury; 30% axonal loss can be detected.  **C:** Fourteen days after crush injury; maximal (75%) axonal loss is seen. Note the central loss of Thy1-CFP-labeled axons.

In the Thy1-CFP mice with crush injury, the proportion of axonal cell loss increased gradually and steadily over time compared to the left eye (Figure 1A), from 30% on day 4 (Figure 1B) to 60% on day 7 and to 75% (maximum) on day 14 (Figure 1C). No change was noted between day 14 and day 21 (75%–77% cell loss, Table 1). No damage to the axons of the optic nerve was detected in the control eyes (Figure 1A). Hoescht staining revealed direct loss of oligodendrocytes (Table 1) to a lesser degree than the reduction in CFP expression at same time point (Figure 1). The early downregulation of CFP appeared to be indicative of oligodendrocyte dysfunction, followed later by cell death.

**Table 1:** The crush model: Loss of cells in the retina and optic nerve over time.

** ** ** ** ** **			**4 days (n=10) % (**±**SD)**	**7 days (n=10) % (**±**SD)**	**14 days (n=10) % (**±**SD)**	**21 days (n=10) % (**±**SD)**
Retina ** **	Thy1-CFP mice (n=40)	CFP	27.4 (±8.7)	58.6 (±11.3)	77.0 (±6.1)	77.4 (±7.4)
		Hoechst	17.2 (±7.6)	47.5 (±5.1)	69.1 (±2.4)	69.5 (±3.3)
Optic nerve	CNPase-GFP mice (n=40)	GFP	28.7 (±6.3)	37.2 (±5.2)	45.2 (±11.6)	52.3 (±8.6)
		Hoechst	31.6 (±6.9)	43.7 (±3.4)	47.7 (±4.3)	51.1 (±5.3)

Comparison of cell loss in the retina and optic nerve in crush injury, by fluorescence labeling of the transgenic mice (CFP or GFP) versus nuclei staining (Hoechst), at different time points. Abbreviations: Thy1, a surface glycoprotein (Thy1); cyan fluorescence protein (CFP); 2', 3′-cyclic nucleotide 3′-phosphodiesterase enzyme (CNPase); green fluorescence protein (GFP); standard deviation (SD).

**Figure 2:**
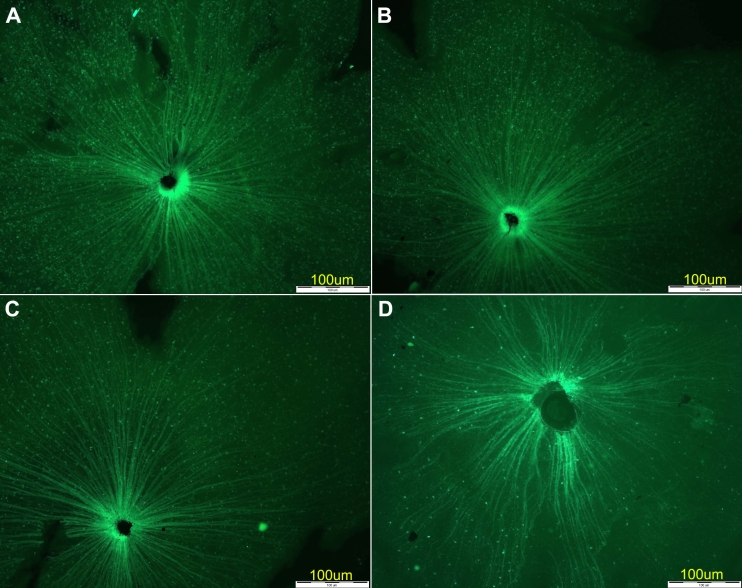
RGC loss in flat-mounted retinas of Thy1-CFP mice at various intervals after crush injury. **A:** Control retina; no damage is detected. **B:** Four days after crush injury; approximately 20%–30% RGC cell loss was detected. **C:** Seven days after crush injury; 50% RGC loss was detected. **D:** Fourteen days after crush injury; maximal 75% RGC loss can be detected. Note the diffuse loss of the labeled cells.

RGC quantification in the flat-mounted retinas (Table 1) revealed a 17%–27% cell loss on day 4 after crush injury (Figure 2B) compared to the control eyes (Figure 2A). This loss increased to 50% on day 7 (Figure 2C) and to 75% (maximum) on days 14 (Figure 2D) and 21.

#### CNPase-GFP transgenic mice

**Figure 3:**
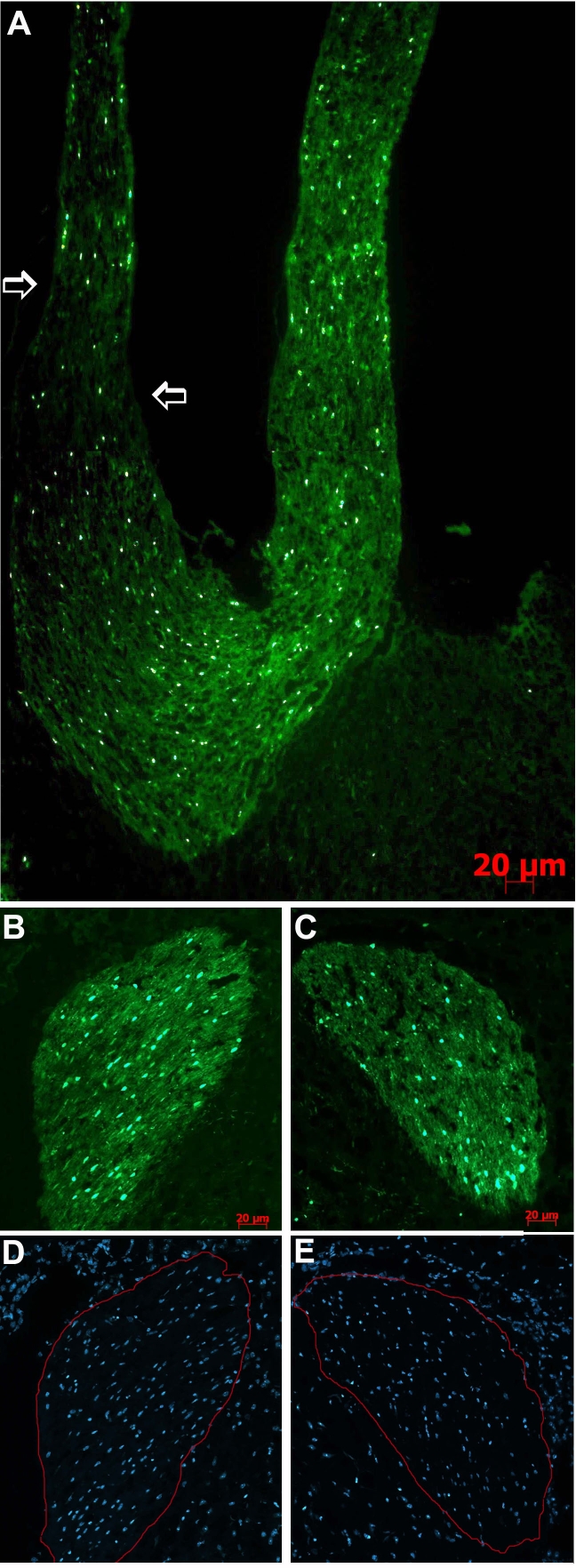
Oligodendrocyte cell loss in chiasm and optic nerves of CNPase-GFP mice at various intervals after crush injury. **A:** Fourteen days after crush injury; approximately 50% oligodendrocyte loss in right optic nerve (white arrow) as compared to the left optic nerve. Note the thin right optic nerve and the reduced fluorescence signal in the right axonal fibers crossing the chiasm toward the contralateral lateral geniculate body. **B** Ipsilateral (right) and **C** contralateral (left) lateral geniculate body (LGB) with 25% oligodendrocyte cell loss in the latter. **D** and **E**: Hoechst staining of both LGB can be seen, with 20% cell loss contralateral to the injured nerve (same magnification, 10X).

Histological study of the samples from the GFP-labeled mice showed 30% oligodendrocyte cell loss on day 4 after crush injury. This rate increased to 40% on day 7 and to a maximum of 50% on days 14 and 21 compared to the untreated eyes (Table 1). The fluorescence signal from the axonal fibers originating from the right injured nerves was lower than that from the left (untreated) nerves (Figure 3A). At that time point (14 days), signs of damage could also be seen in the contralateral lateral geniculate body of the crushed nerve (Figure 3C,E), which had 25% fewer cells than its counterpart in the untreated nerve (Figure 3B,D).

### rAION model

#### Thy1-CFP transgenic mice

**Figure 4:**
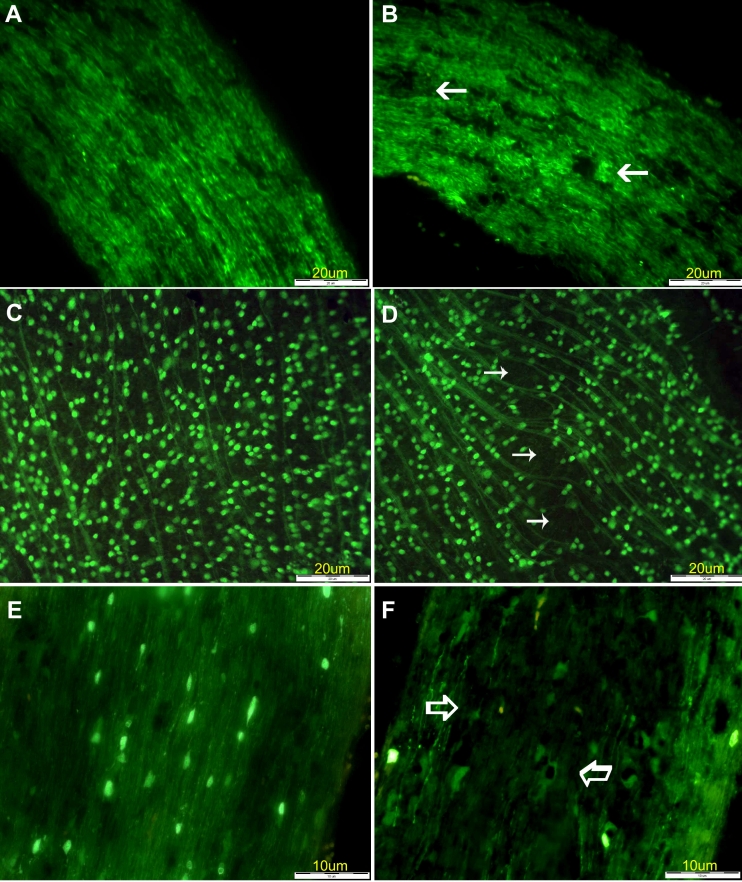
Retinal and optic nerve findings at various intervals after rAION induction. **A:** Control optic nerve from Thy1-CFP transgenic mouse; no axonal loss is detected. **B:** Histologicial section of the optic nerve at 21 days after rAION induction; arrows point to moderate axonal loss (maximal, 25%). **C:** Flat-mounted retina from control (untreated) eye of same mouse showing normal density of RGC nuclei. **D**. Flat-mounted retina from study eye showing RGC loss, as compared to control (**C**). **E:** Histological section of control (untreated) optic nerve from CNPase-GFP transgenic mouse. Note the complete myelinization and number of oligodendrocyte nuclei. **F:** Twenty-one days after rAION induction, maximal (20%–30%) oligodendrocyte loss is demonstrated.

With the method used in this study, no significant axonal loss was detected at 4, 7, and 14 days after rAION induction. Examination at 21 days yielded a maximal axonal loss of 25% (Figure 4B). No axonal loss could be detected in the untreated control eyes (Figure 4A). Accordingly, RGC quantification in the flat-mounted retinas showed no significant cell loss at any time before 21 days, when a 25% decrease was noted compared to the untreated control eyes (Figure 4C,D; Table 2).

**Table 2:** Detection of cell loss in the retina and optic nerve in both models of injury, by fluorescence label versus nuclei staining (Hoechst) and TUNEL.

**Models of injury**				**CRUSH n=40** **% (**±**SD)**		**rAION** **n=40 % (**±**SD)**	
Retina	RGC loss	Thy1-CFP mice (n=20)	CFP 21 d (n=5)	77.4 (±7.4)		25.3 (±9.5)	
			Hoechst 21d (n=5)	69.5 (±3.3)		22.9 (±14.7)	
		TUNEL	apoptotic 1d/3d (n=5)	25%	40%	5%	20%
Optic nerve	OLG loss	CNPase-GFP mice (n=20)	CNPase 21d (n=5)	52.3 (±8.6)		27.4 (±4.6)	
			Hoechst 21d (n=5)	51.1 (±5.3)		20.3 (±7.2)	

Comparison of cell loss in the retina and optic nerve in both models of injury, by fluorescence (CFP or GFP) versus nuclei staining (Hoechst), at day 21. Our finding of greater RGC loss and axonal damage in the Thy1-CFP mice subjected to crush injury compared to the Thy1-CFP mice subjected to rAION induction indicated that the crush injury caused more damage than rAION. In both models, the damage progressed within days, and loss of oligodendrocytes could be detected on day 21, more in the crush model. We were also able to show that the optic nerve injury, induced in both crush and rAION models, led to anterograde RGC apoptosis. Apoptosis assays (TUNEL) at days 1 and 3 indicate apoptotic mechanism of the retinal ganglion cell loss, increasing towards day 3. Maximal RGC loss (irreversible loss) calculated for day 21 showed 77.4% in the crush model, and 25.3% for the rAION model.

#### CNPase-GFP transgenic mice

No significant oligodendrocyte loss was detected before 21 days, when findings yielded a decrease of 20%–30% compared to controls (Figure 4E,F; Table 2).

### Apoptosis assay

**Figure 5:**
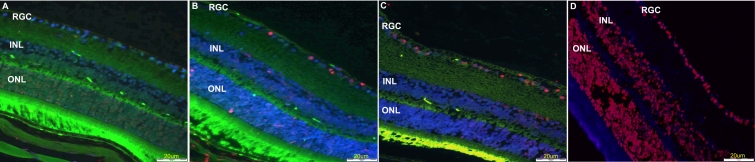
Apoptosis assay: retina. **A**: Control (untreated) eye; no staining for apoptosis was detected. **B:** Three days after rAION induction, TUNEL staining yielded positive cells along the retina **C:** Three days after crush injury, more positive cells were detected. **D**: Retinal section served for positive control of the apoptosis assay.

After crush injury, TUNEL-positive nuclei were detected mainly in the retinal sections, in the RGC layer. The estimated rate of labeled cell bodies was 25% at 1 day after injury and 40% after 3 days (Figure 5C). TUNEL-labeled cells were also found in the retinal sections after rAION induction, but at lower levels: an estimated 5% on day 1 and 20% on day 3 (Figure 5B; Table 2). At 7 days following both crush and rAION induction, there were no detectable TUNEL-positive cells in the retina (data not shown). No TUNEL-positive cells were observed in the retinal sections of the untreated eyes (Figure 5A).

**Figure 6:**
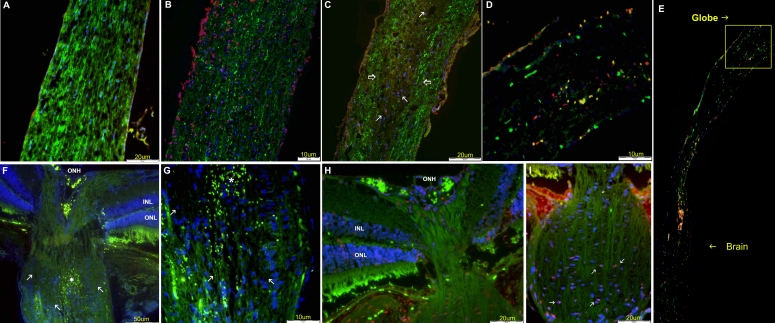
Apoptosis assay: optic nerve, after crush and rAION. **A**: Normal optic nerve; no apoptotic cells are detected. **B:** Positive control. **C:** TUNEL-positive (red) cells along the optic nerve 3 days following crush injury of wild type mice. **D**: TUNEL staining of optic nerve of GFP-CNPase mice 3 days following crush showing positive staining. Oligodendrocytes apoptotic cells are yellow (green for GFP and red represent positive staining for apoptosis), suggesting that oligodendrocytes in the optic nerve proximal to the globe undergo apoptosis 3 days following induction of crush injury. **E:** The whole optic nerve of same GFP-CNPase mice (**D**) is shown. **F**: Optic nerve head, 3 days following crush injury; showing hemorrhage (asterisk), immediately posterior to the globe. **G**: Same damaged area at higher magnification, demonstrating loss of oligodendrocytes and focal hemorrhagic area. **H**: Optic nerve head 3 days following rAION induction, showing preserved architecture of retina and intraocular optic nerve, without apoptotic cells. **I**: Same nerve as **H**, 3 days after rAION, demonstrating the anterior segment of the optic nerve behind the globe. Note few TUNEL-positive cells (red staining, arrows) at the center of the anterior optic nerve.

TUNEL-positive nuclei in the optic nerves were detected mainly following crush injury (Figure 6C). Staining for TUNEL of GFP-CNPase axons revealed a double labeling of cells, confirming that the oligondendrocyte loss follows the apoptosis pathway (Figure 6D,E). TUNEL-labeled cells were detected in the damaged area behind the globe one day after injury, with a few apoptotic cells further along the nerve on day 3 (Figure 6). Bleeding could be seen in the center of the damaged area, in addition to oligodendrocyte loss (Figure 6F,G). Following rAION induction, a few apoptotic cells were detected anteriorly in the optic nerve, just behind the globe, with a peak on day 3 after induction of injury (Figure 6H,I). No apoptotic cells were detected in the control left eye (Figure 6A).

## Discussion

This study demonstrates the progression of axonal involvement and demyelination along the optic nerve following crush injury or rAION. We used 2 murine models of injury to compare different intensities of damage, and 2 types of transgenic mice to compare the pathophysiologic effects at different levels.

Given that Thy1 in the retina is expressed selectively in the axons and cell bodies of RGCs [25], fluorescence labeling makes it possible to directly identify the cells microscopically in the retinal ganglion layer and to perform a semiquantitative analysis (as almost all the cells are labeled) [14]. Because this method eliminates the need for further histological processing, it appears to be advantageous to other dye-labeling methods which may harbor technical difficulties [14]. Its use is supported by the compatible results found in the present study between the change in *Thy1* mRNA retinal expression on molecular analysis and RGC reduction on histological analysis in Thy1-CFP-labeled mice after rAION induction.

Our finding of greater RGC loss and axonal damage in the Thy1-CFP mice subjected to crush injury compared to the Thy1-CFP mice subjected to rAION induction indicated that the crush injury caused more damage than rAION. In both models, the damage progressed within days, and axonal loss could be detected up to the contralateral lateral geniculate body–the first synapsing nucleus for axons originating in the RGCs on their way to the visual cortex (Figure 3B,C). We were also able to show that the axonal damage led to anterograde RGC loss within days, reaching maximum (irreversible loss) between days 14 and 21 (Table 2).

After rAION induction, RGC and axonal loss reached 25% of baseline at 21 days. Molecular analysis with real-time PCR revealed Thy1 levels of expression declined by 27% at the same time point (data from previous studies not shown).

By contrast, the more intense damage induced by the crush injury led to a 27% decrease in RGC level by 4 days after injury, and a 77% RGC loss by 14 days, with no further retinal damage. This finding is consistent with the 20% loss in mRNA Thy1 expression reported by Schlamp et al. [20]. Huang et al. [19] noted a greater (50%) decrease in Thy1 gene expression at 5 days after crush injury, but they used a rat model, so the difference could be attributable to species variation.

We found the same amount of axonal loss and RGC loss at all time points after crush injury. This finding indicates that axonal loss may occur concurrently with RGC loss after crush injury.

Our second group of transgenic mice expressed the CNPase gene promoter linked to GFP and was used to evaluate the changes in oligodendrocytes in response to the two intensities of optic nerve injury. The findings in this group complemented those in the first group, clearly demonstrating the time and order of events. We noted a greater (30%) decrease in fluorescence in the optic nerve (oligodendrocytes and myelin) than in the retina (RGCs) at the early stage of injury (4 days), indicating that the optic nerve is affected simultaneously if not primarily. This agrees with studies of other demyelinating diseases wherein loss of oligodendrocytes and myelin apparently led to axonal loss [15,26,27].

TUNEL staining supported these findings, showing that in both models of injury, apoptosis occurred shortly after the damage was induced, simultaneously in the optic nerve and retina. There was more apoptosis after crush than after rAION injury, because rAION is a more gentle injury. Positive apoptotic cells were detected 3 days after damage. By contrast, Berkelaar et al. [28] first detected apoptotic cells only 5 days after damage. However, they induced intracranial lesions 8–9 mm from the eye whereas, in our rAION model, damage was induced to the head of the optic nerve, and in our crush model, proximal to the globe of the eye. This may explain the earlier apoptotic pathway in our study. In line with our study results are the long interval between the axonal injury and RGC death in the earlier report [28] and the different times of onset of the massive RGC loss with optic nerve lesions near or far from the eye. We speculate that the more severe crush injury may have led to immediate apoptosis or even damage to the area by necrosis. Further studies are needed to clarify this issue. The presence of a feedback effect is also still unclear, with damage to the axons causing oligodendrocyte loss. Clearly, axonal damage places stress on the cell bodies in the retina.

Better understanding of the pathophysiology of optic nerve damage might aid the development of targeted neuroprotective agents.
